# Protein Expression Profiles Characterize Distinct Features of Mouse Cerebral Cortices at Different Developmental Stages

**DOI:** 10.1371/journal.pone.0125608

**Published:** 2015-04-27

**Authors:** Haijun Zhang, Yoko Kawase-Koga, Tao Sun

**Affiliations:** 1 Department of Cell and Developmental Biology, Weill Medical College of Cornell University, New York, New York, United States of America; 2 Department of Genetic Medicine, Weill Medical College of Cornell University, New York, New York, United States of America; 3 Department of Oral and Maxillofacial Surgery, The University of Tokyo Hospital, Tokyo, Japan; The Walter and Eliza Hall of Medical Research, AUSTRALIA

## Abstract

The proper development of the mammalian cerebral cortex requires precise protein synthesis and accurate regulation of protein expression levels. To reveal signatures of protein expression in developing mouse cortices, we here generate proteomic profiles of cortices at embryonic and postnatal stages using tandem mass spectrometry (MS/MS). We found that protein expression profiles are mostly consistent with biological features of the developing cortex. Gene Ontology (GO) and KEGG pathway analyses demonstrate conserved molecules that maintain cortical development such as proteins involved in metabolism. GO and KEGG pathway analyses further identify differentially expressed proteins that function at specific stages, for example proteins regulating the cell cycle in the embryonic cortex, and proteins controlling axon guidance in the postnatal cortex, suggesting that distinct protein expression profiles determine biological events in the developing cortex. Furthermore, the STRING network analysis has revealed that many proteins control a single biological event, such as the cell cycle regulation, through cohesive interactions, indicating a complex network regulation in the cortex. Our study has identified protein networks that control the cortical development and has provided a protein reference for further investigation of protein interactions in the cortex.

## Introduction

Early development of the mammalian cerebral cortex is a dynamic and complex process. Neural progenitors undergo symmetric and asymmetric division to expand the progenitor population at the ventricular zone and subventricular zone at embryonic stages [1–7]. Neural progenitors then differentiate into distinct types of neurons that migrate into the cortical plate at the perinatal stage [8,9]. This developmental process requires precise protein expression in different cells in a temporal manner. The dynamic changes of protein expression profiles in the cortex at different developing stages are not well understood.

The technique development of tandem mass spectrometry (MS/MS) has made high throughput proteomic analysis possible to reveal cell type- and tissue-specific protein expression profiles [10]. The protein, as the final product of a corresponding gene, excels its function and controls behaviors of cells. Thus, levels of protein expression in the cortex are crucial for controlling proliferation and differentiation of neural progenitors. Proteomics can be used to quantify overall protein expression levels in specific tissues, identify differentially expressed proteins and ultimately reveal protein expression signatures.

Previous reports have attempted to perform brain proteomic profiles mainly using two-dimensional electrophoresis-based technology [11–16]. Systemic evaluation of proteomes of the developing mouse cortex has not yet been well studied. In this study, to identify protein expression profiles in the developing mouse cortex, we quantified protein expression levels in cortices at embryonic day 13.5 (E13.5), E15.5 and postnatal day 1 (P1) using MS/MS. We found that the protein expression signatures reflect features of temporal development of the cortex. Bioinformatics tools further revealed differentially expressed proteins in cortices at different stages and identified interaction networks of proteins. Our proteome report of developing mouse cortices has revealed the signature of protein profiles in the cortex at different developmental stages and provided novel protein interaction networks for further understanding the brain function.

## Materials and Methods

### Proteomic sample preparation and processing

The dorsal cortical regions from brains of C57/BL6 mice (Charles River) at E13.5, E15.5 and P1 were dissected. At each developmental stage, protein extraction from 3 brains were pooled, 9 brains were used for 3 runs. Totally 27 brains were used for all three stages. Cortical tissues were washed twice with cold PBS and homogenized in homogenization buffer [15 mM Tris-HCl (pH 7.7), 0.5 mM PMSF, 0.25 M sucrose, 15 mM NaCl, 1.5 mM MgCl2, 2.5 mM EDTA (pH 8.0), 1 mM EGTA (pH 8.0), 25 mM NaF, 2 mM NaPPi, Protease inhibitor Cocktail Complete (Roche)]. For each stage per experiment, 50 μg of total protein were transferred into proteomics buffer [20 mM Tris-HCl (pH 8.0), 6 M urea, 2 M thiourea, 4% CHAPS, 1 mM EDTA (pH 8.0), 1 mM PMSF, Protease inhibitor Cocktail Complete, 0.2 mM Na3VO4, 1 mM NaF] and fractionated by SDS-PAGE. Protein gels were stained with Coomassie Blue (PIERCE) for 24 h. Gel fragments were cut and subjected to trypsin digestion. The resultant peptides were analyzed using liquid-chromatography (LC)-tandem mass spectrometry (MS/MS). In detail, gel slices were cut along the entire length of the gel, chopped into 12 sections. Each section was reduced with 10 mM dithiothreitol (Calbiochem, San. Diego, CA) and alkylated with 100 mM iodoacetamide (Sigma, St Louis, MO). Gel digestion was performed with the sequence grade modified trypsin (Promega, Fitchburg, WI) in 50 mM ammonium bicarbonate at 37°C overnight. The peptides were extracted twice with 1% trifluoroacetic acid in 50% acetonitrile aqueous solution for 30 min, according to published protocols [17].

For LC-MS/MS analysis, each digestion product was separated by a 60 min gradient elution at a flow rate of 250 nl/min with a Dionex capillary/nano-HPLC system and analyzed by a QSTAR XL mass spectrometer (Applied Biosystems) using information-dependent, automated data acquisition. The analytical column was a homemade 75 μm i.d. x 15 cm reversed-phase C-18 resin (300 Å, 5 μm, Varian, Lexington, MA) column. Mobile phase A consisted of 0.1% formic acid, and mobile phase B consisted of 100% acetonitrile and 0.1% formic acid. The gradients for solvent B increased from 0% to 55% in 30 min and then from 55% to 80% in 10 min. BioWorks 3.3.1 (Thermo-Fisher, San Jose, CA) was used to convert the MS/MS spectra of each LC-MS/MS run from RAW file to DTA file. The DTA files were searched against the mouse IPI database using an in-house Mascot searching algorithm. The search parameters were as following: maximum of 1 missed trypsin cleavages, cysteine carbamidomethylation as the fixed modification, methionine oxidation as the variable modification. The maximum error tolerance was 1 Da for MS/MS. When the Mascot score was more than 35, proteins were designated as “hits”. When several proteins matched the same sets of peptides, only the protein with the greater percentage of coverage was selected.

The “protein score” and “protein matches” were acquired by MS/MS report. The protein score indicates the combined scores of all recorded mass spectra which can be matched to amino acid sequences within that protein. A higher score reflects a more confident match. The protein matches, indicating the count of interpretable MS/MS spectra per protein, were used to quantify protein hits, and this number of peptides identified for each protein is largely proportional to the quantity of the protein in the sample.

### Ethics Statement

Animal use was overseen by the Animal Facility and approved by the IACUC (protocol number: 2011–0062) at the Weill Cornell Medical College.

### Proteomic data analyses

Because the proteomic data may be derived from different amounts of original proteins, or processed at different times, these data were normalized prior further analyses to reduce biased results. The trimmed mean normalization was used, because it is more robust to resist the outlier of the data, compared to the mean normalization. Four percent trimmed mean was used to normalize the data and the trimmed mean of 100 was set for each sample. In detail, we normalized the value of each sample by removing top and bottom 2% values, calculated the mean of the remaining values, divided each value in a sample by the trimmed mean and multiplied them by 100. After normalization, the remaining “0” value was assigned to a small value 1 to facilitate subsequent data comparison. The normalized value was log2-transformed and was used as the protein expression value for further analysis. After log2-transformation, upregulated and downregulated proteins were recognized as changing of expression by the same amplification. To show the frequency distribution of identified proteins by MS/MS, these proteins were assigned into different bins (from < -2 to > 8) by their protein expression value. The number of protein ID in each bin was summed as the frequency of this bin. To show the proportional frequency distribution of identified proteins by MS/MS, these proteins were assigned into different bins (from < -2 to > 8) by their protein expression value, and the number of protein ID in each bin divided by the number of the total protein ID was summed as the proportional frequency of this bin.

Hierarchical clustering analysis was conducted by R 2.15.2. The log2-transformed value of each protein, e.g. the protein expression value of this protein, was used for the differential expression analysis. Differentially expressed proteins between developmental stages were identified by comparing protein expression values of each protein between these two stages. The statistical significance was defined as *P* < 0.05 of Student’s *t* test.

### Protein functional pathway analyses

Functional analyses were performed using Database for Annotation, Visualization and Integrated Discovery (DAVID, http://david.abcc.ncifcrf.gov/home.jsp). The list of differentially expressed proteins between E13.5 and E15.5, or E13.5 and P1 was uploaded and analyzed using the default settings of EASE *P* value < 0.1. The whole mouse genome was used as a background list as used in other brain proteomic studies [16]. Proteins in the list were annotated by the biological process, cellular component, and molecular function of the Gene Ontology (GO) and the KEGG canonical pathway database. The functional association network of differentially expressed proteins was constructed by STRING, a database of known and predicted interactions, including direct physical interactions and indirect functional interactions (http://string-db.org/).

## Results

### Generating protein expression profiles in the developing mouse cerebral cortex

**Fig 1:**
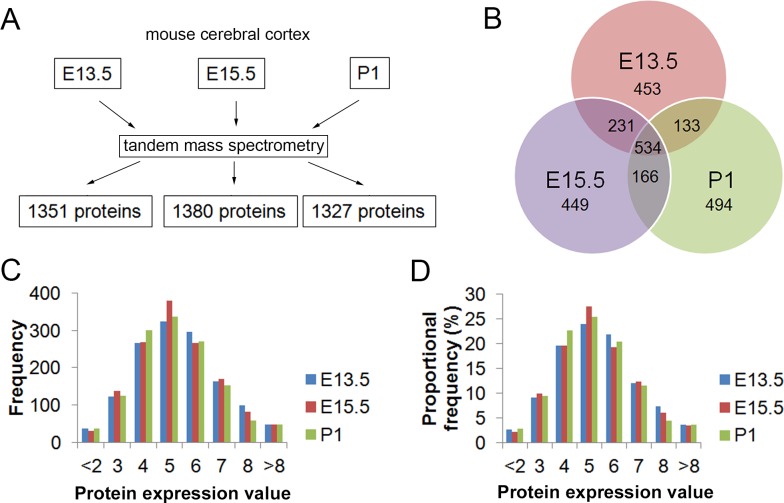
Generate protein expression profiles of the developing mouse cerebral cortex using tandem mass spectrometry (MS/MS). (A) A flow chart showing protein identification process using MS/MS. Protein expression was analyzed by MS/MS. To identify proteins with significant expression levels in the cortex, the ‘protein score’ with the threshold > 35 was applied. As a result, 1351, 1380, and 1327 proteins were identified in cortices at E13.5, E15.5 and P1, respectively. (B) Diagram summarizing the pattern of proteins found in mouse cortices at three developmental stages. Each sphere indicates proteins expressed at one stage. The overlapping part of the spheres indicates proteins identified at both stages. The numbers of proteins identified are labeled accordingly. (C) Frequency distribution of identified proteins by MS/MS. Identified proteins by MS/MS were assigned into different bins (from < -2 to > 8) by their protein expression value (see details in Materials and Methods for the definition of protein expression value). Briefly, protein matches were normalized and log2-transformed to generate protein expression value. The number of protein ID in each bin was summed as the frequency of this bin. (D) Proportional frequency distribution of identified proteins by MS/MS. Identified proteins by MS/MS were assigned into different bins by their protein expression value and the number of protein ID in each bin divided by the number of the total protein ID was summed as the proportional frequency of this bin.

In the developing cerebral cortex, proliferation and differentiation of neural progenitors require rapid protein synthesis. To reveal what proteins are highly expressed at different developmental stages, we generated protein expression profiles in the mouse cerebral cortex. Total proteins from the dorsal cortical region of mouse brains were extracted at E13.5, E15.5 and P1. Protein expression was analyzed by MS/MS (Fig 1A). To identify proteins with significant cortical expression levels, the ‘protein score’ with the threshold > 35 was applied. As a result, 1351, 1380, and 1327 proteins were identified in E13.5, E15.5 and P1 cortices, respectively (Fig 1A). In total, 1966 proteins were detected in E13.5 and E15.5 cortices, and 2011 proteins detected in E13.5 and P1 cortices, and 2460 proteins detected in cortices of all three stages (S1 Table). Among 2460 proteins, 534 proteins were found in all three stages, suggesting that they may play a conserved role in cortical development (Fig 1B). There were 765 proteins found in both cortices of E13.5 and E15.5, 667 proteins in both cortices of E13.5 and P1, and 700 in both cortices of E15.5 and P1 (Fig 1B). There were more proteins detected in both cortices of E13.5 and E15.5 than those of E13.5 and P1, indicating a higher similarity of protein expression profile in embryonic cortices than postnatal cortices. Moreover, cortices of E15.5, compared to those of E13.5, shared more proteins with cortices of P1, suggesting a temporal regulation of the protein expression along the process of cortical development.

### Proteomic profiles reflect features of embryonic and postnatal cortices

To quantify protein expression levels detected by MS/MS, ‘protein matches’ were used, and the values of protein matches were normalized and log2-transformed (see details in Materials and Methods). Patterns of the frequency distribution and proportional frequency distribution of values of protein expression were similar at each stage, suggesting that protein expression levels are comparable among three stages (Fig 1C and 1D).

Furthermore, a hierarchical tree was generated by the “hclust” function in R based on the values of protein expression among samples. Samples with similar protein expression patterns were grouped together and were linked by a series of branches of a clustering tree. Proteins detected in cortices of E13.5 and E15.5 were clustered into the same branch, which further indicating that the proteomic profiles are similar between the embryonic stages, compared to those between the embryonic-postnatal stages (S1 Fig).

**Fig 2:**
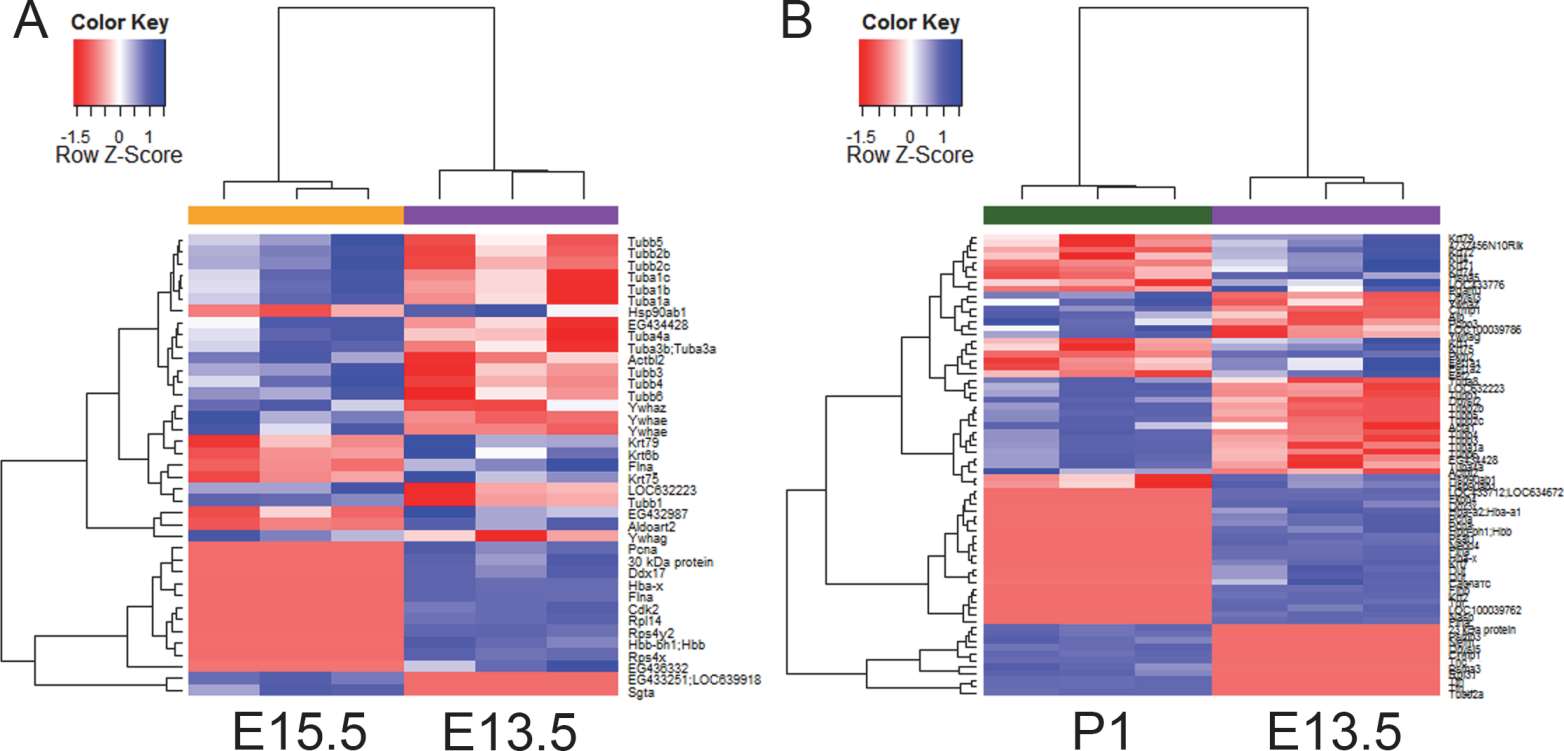
Hierarchical clustering for differentially expressed proteins in cortices at three stages. (A) Differentially expressed proteins between E13.5 and E15.5. (B) Differentially expressed proteins between E13.5 and P1. Protein expression value was used to represent the individual protein expression and was used in the hierarchical analysis.

We next detected differentially expressed proteins, which are either significantly highly expressed or are only detected, at one developmental stage, by comparing values of protein expression between two stages. Among 1966 proteins that were detected in cortices at either E13.5 or E15.5, 39 proteins (2%) were differentially expressed; and among 2011 proteins detected in cortices at either E13.5 or P1, 71 proteins (3.5%) were differentially expressed (Fig 2 and S2 Table). Our results indicate a more similar proteomic profile between embryonic stages than that between the embryonic-postnatal stages.

### Functional pathways of cortical proteins detected by proteomics

**Fig 3:**
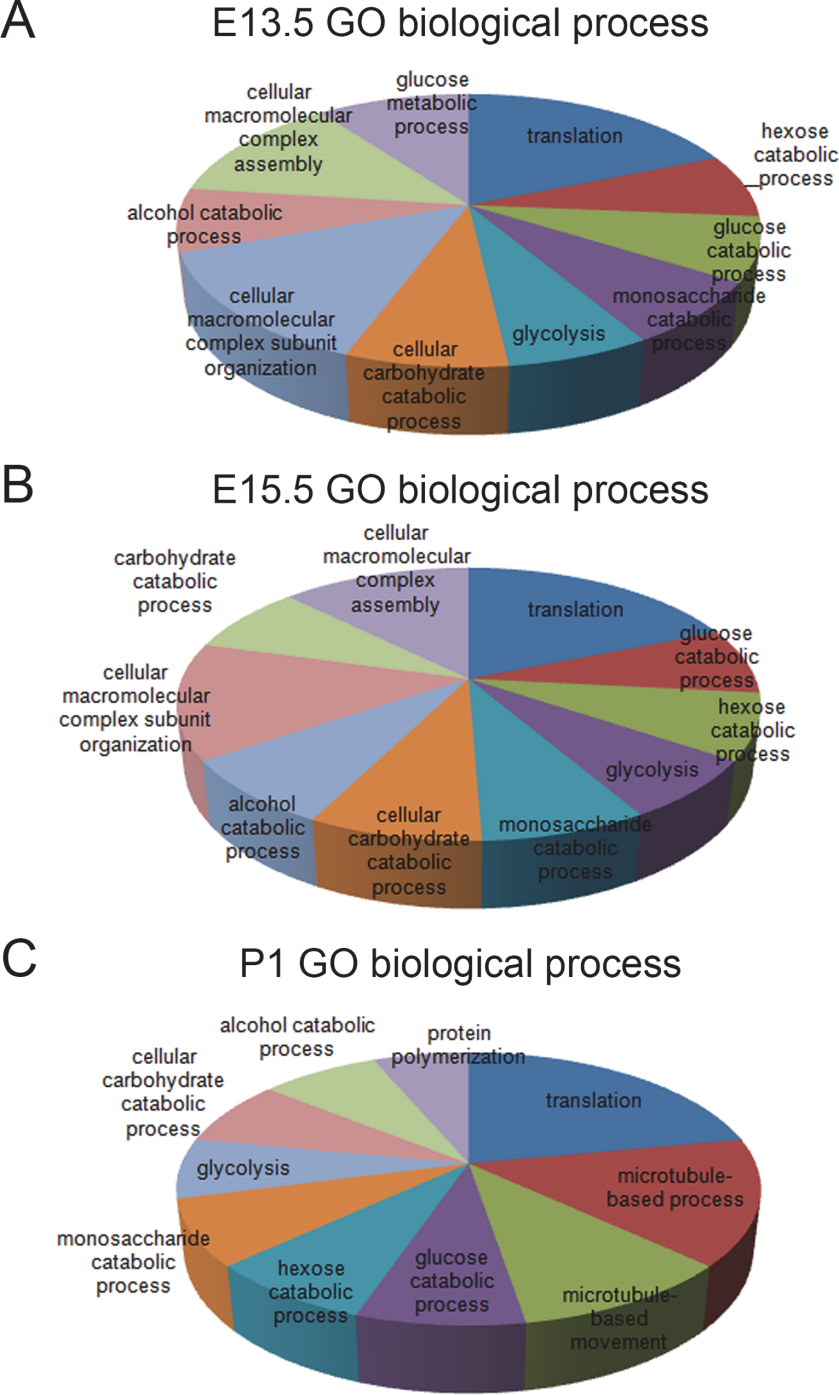
Gene Ontology (GO) biological process analyses for proteins expressed in cortices at E13.5 (A), E15.5 (B), and P1 (C). The default setting for Database for Annotation, Visualization and Integrated Discovery (DAVID) was used to perform the analysis. Only top 10 enriched terms were shown in the pie chart.

We next analyzed functions of detected proteins by proteomics in cortices of three different developing stages using DAVID [18,19]. Proteins related to translation and metabolic process were enriched among all three stages as detected by Gene Ontology (GO) analyses. Moreover, proteins involved in cellular macromolecular complex assembly were enriched in cortices of E13.5 and E15.5, while proteins functioning in polymerization and microtubule-based process were highly detected in P1 cortices (Fig 3 and S3 Table).

**Fig 4:**
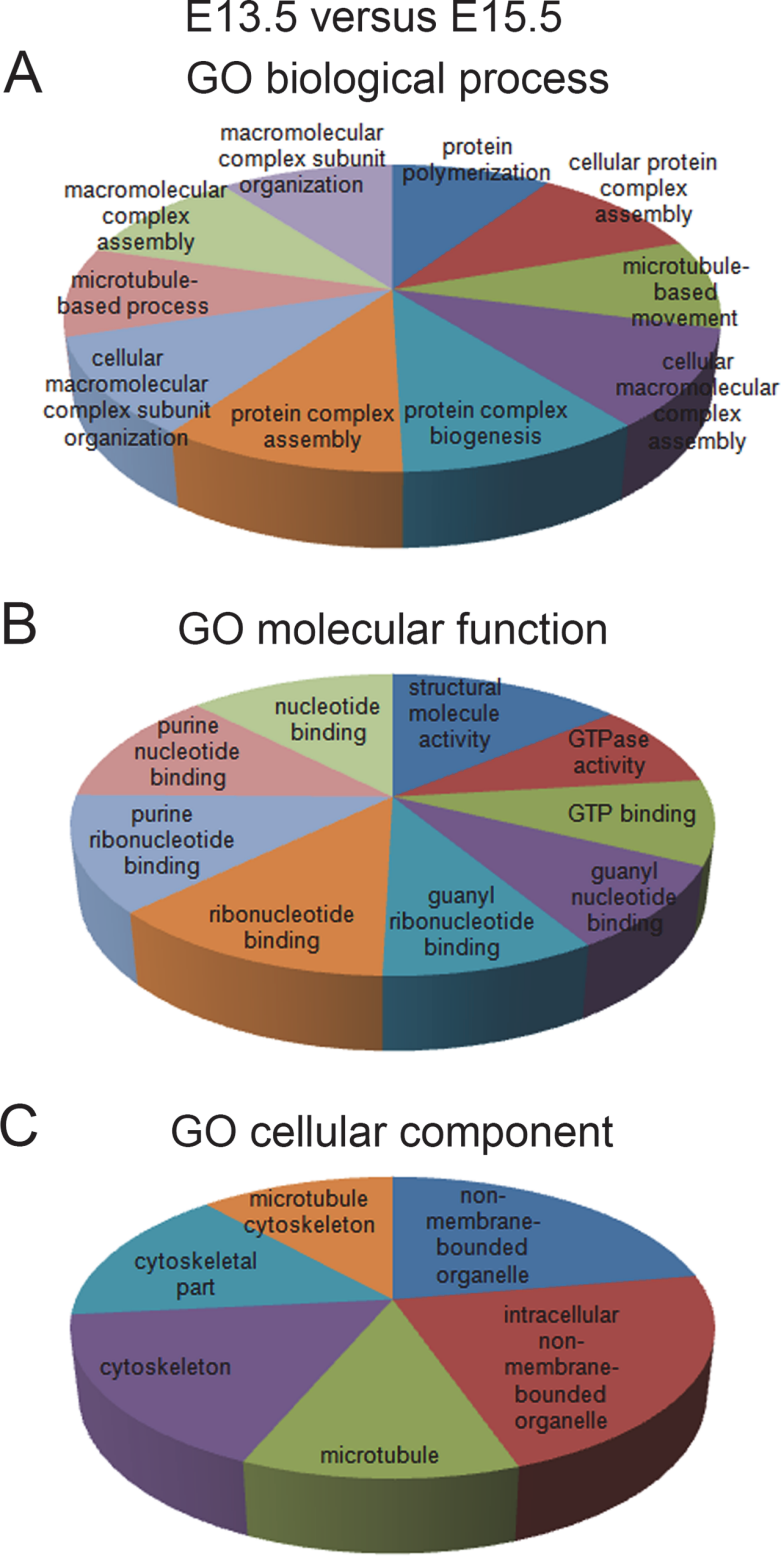
Analyses of biological process (A), molecular function (B), and cellular component (C) for proteins differentially expressed in cortices between E13.5 and E15.5. The default setting for Database for Annotation, Visualization and Integrated Discovery (DAVID) was used to perform the analysis. Only top 10 enriched terms were shown in the pie chart.

We further categorized functions of differentially expressed proteins through DAVID GO and canonical KEGG pathway analyses [20,21]. Similar to the overall protein functional grouping, differentially expressed proteins involved in macromolecular complex organization and microtubule formation showed enriched expression in cortices of E13.5 and E15.5 (Fig 4 and S4 Table). A few proteins that regulate the cell cycle and metabolism were also detected, suggesting important developing events at E13.5 and E15.5 (S4–S6 Tables). CDK2, PCNA and 14-3-3 isoforms were identified in the cell cycle pathway, among differentially expressed proteins in cortices between E13.5 and E15.5; and PCNA and 14-3-3 isoforms were identified in cortices between P1 and E13.5 (S2 Fig). Interestingly, proteins regulating the gap junction, such as isoforms of α- and β-tubulins, were detected among differentially expressed proteins using the KEGG pathway analysis (S6 Table).

Furthermore, to illustrate functions of differentially expressed proteins detected at three stages (detailed expression levels of these proteins were shown in S2 Table), we selected 24 proteins and manually searched the PubMed database to explore their roles in the nervous system (Table 1). Functions of these proteins suggest that proteomics has identified proteins involved in important biological events during cortical development. For example, Flna (filamin A) was detected to show high expression in E13.5 cortices (S2 Table), and Flna was reported to regulate neural progenitor proliferation and cortical size [22]. TUBB3 (tubulin β-3 chain, also called Tuj1) showed increased expression during cortical development (S2 Table), and was shown to play a critical role in proper axon guidance and maintenance [23]. Nefm (neurofilament medium polypeptide) was detected in the P1 cortex (S2 Table), and was reported to play a role in intracellular transport to axons and dendrites [24]. Thus, our proteomic analysis has identified differentially expressed proteins involved in multiple functions in the developing cortex.

**Table 1:** Proteins differentially expressed during the cortical development and their potential functions in the nervous system.

Gene Symbol	Gene name	Function in the nervous system
Flna	Filamin A	Regulate neural progenitor proliferation and cortical size [22]
Cdk2	Cell division protein kinase 2	Cdk2 is critical for proliferation and self-renewal of neural progenitor cells in the adult subventricular zone [31]
Pcna	Proliferating cell nuclear antigen	Proliferation marker [32]
Tubb2b	Tubulin beta-2B chain	Mutation in the β-tubulin gene TUBB2B associated with complex malformation of cortical development and deficits in axonal guidance [33]
Ywhae	Tyrosine 3-monooxygenase/tryptophan 5-monooxygenase activation protein, epsilon polypeptide; 14-3-3 protein epsilon	Deletion of YWHAE is found in a patient with periventricular heterotopias and pronounced corpus callosum hypoplasia [34]
Tubb3	Tubulin beta-3 chain	TUBB3 plays a critical role in proper axon guidance and maintenance [23]
Tubb1	Tubulin, beta 1	Neurogenesis [35]
Tuba1a	Tubulin alpha-1A chain	Neural-specific α-tubulin isoform whose expression is restricted to the developing and regenerating nervous system [36]
Tubb5	Tubulin beta-5 chain	TUBB5 and its disease-associated mutations influence the terminal differentiation and dendritic spine densities of cerebral cortical neurons; mutations in the *TUBB5* cause microcephaly with structural brain abnormalities; deficient mice exhibit schizophrenic behaviors [37,38]
Tuba8	Tubulin alpha-8 chain	Mutation of the variant alpha-tubulin TUBA8 results in polymicrogyria with optic nerve hypoplasia [39]
Ywhag	14-3-3 protein gamma	Downregulated in the schizophrenic cortex [40,41]
Ywhaz	14-3-3 protein zeta/delta	Knockout mice exhibit schizophrenic behaviors [42]
Tnc	Isoform 1 of Tenascin precursor	Neural stem/progenitor cells express 20 tenascin C isoforms that are differentially regulated by Pax6 [43]
Tubb2a	Tubulin beta-2A chain	De novo mutations in the beta-tubulin gene TUBB2A cause simplified gyral patterning and infantile-onset epilepsy [44]
Psat1	Phosphoserine aminotransferase	PSAT1 may be implicated in altered serine metabolism and schizophrenia spectrum conditions [45]
Crmp1	Dihydropyrimidinase-related protein 1	Collapsin response mediator protein 1 mediates reelin signaling in cortical neuronal migration [46]
Nedd4	E3 ubiquitin-protein ligase	E3 ligase Nedd4 promotes axon branching by downregulating PTEN [47]
Dpysl5	Dihydropyrimidinase-related protein 5	This gene encodes a member of the CRMP (collapsing response mediator protein) family thought to be involved in neural development [48]
Nefm	Neurofilament medium polypeptide	Neurofilaments comprise the axoskeleton and functionally maintain neuronal caliber. They may also play a role in intracellular transport to axons and dendrites [24]
Dpysl2	Dihydropyrimidinase-related protein 2	Promotes microtubule assembly and is required for Sema3A-mediated growth cone collapse, and also plays a role in synaptic signaling through interactions with calcium channels [49,50]
Hba-a2	hemoglobin alpha, adult chain 2	Hemoglobin chains are expressed in neurons and are regulated by treatments that affect mitochondria, opening up the possibility that they may play a novel role in neuronal function and response to injury [51]
Eef2	Elongation factor 2	As a biochemical sensor coupling miniature synaptic transmission to local protein synthesis [52]
Cacna1c	calcium channel, voltage-dependent, L type, alpha 1C subunit	Forebrain elimination of cacna1c mediates anxiety-like behavior in mice [53]
Eef1a2	Elongation factor 1-alpha 2	De novo EEF1A2 mutations in patients with characteristic facial features, intellectual disability, autistic behaviors and epilepsy [54]

### Functional networks of differentially expressed proteins in developing cortices

**Fig 5:**
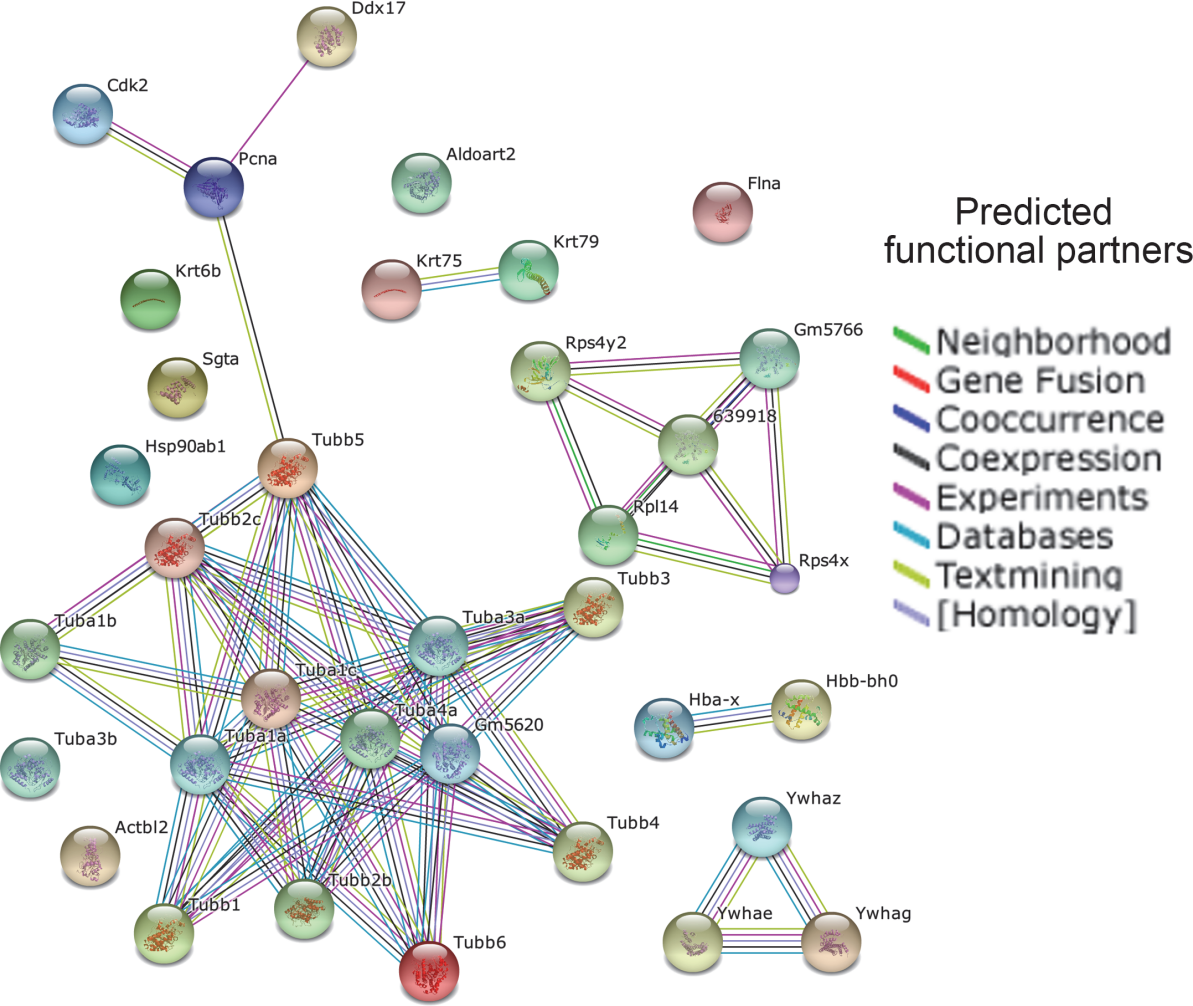
Functional protein association network of differentially expressed proteins in cortices at E13.5 and E15.5. Different line colors represent the types of evidence for the association: green, neighborhood; red, gene fusion; blue, co-occurrence; black, co-expression; purple, experiments; turquoise, database; yellow, text mining; and aqua, homology. The default setting for SPRING 9.1 was used to perform the analysis.

We next analyzed protein-protein interactions among differentially expressed proteins at three developing stages using STRING [25]. The differentially expressed proteins in cortices between E13.5 and E15.5 were detected to form three major clusters, which include 1) microtubule (α- and β-tubulin proteins Tubas and Tubbs, and others), and cell cycle (CDK2 and PCNA), 2) 14-3-3 (Ywhae, Ywhag, and Ywhaz) and 3) ribosome (ribosomal proteins Rps4x, Rpx4y2, and Rpl14). Hemoglobins (Hba-x and Hbb-bh0) and keratins (Krt75 and Krt79) were coupled in their own categories, and other identified proteins such as Aldoart2 and Hsp90ab1 were not incorporated into any sub-network (Fig 5).

**Fig 6:**
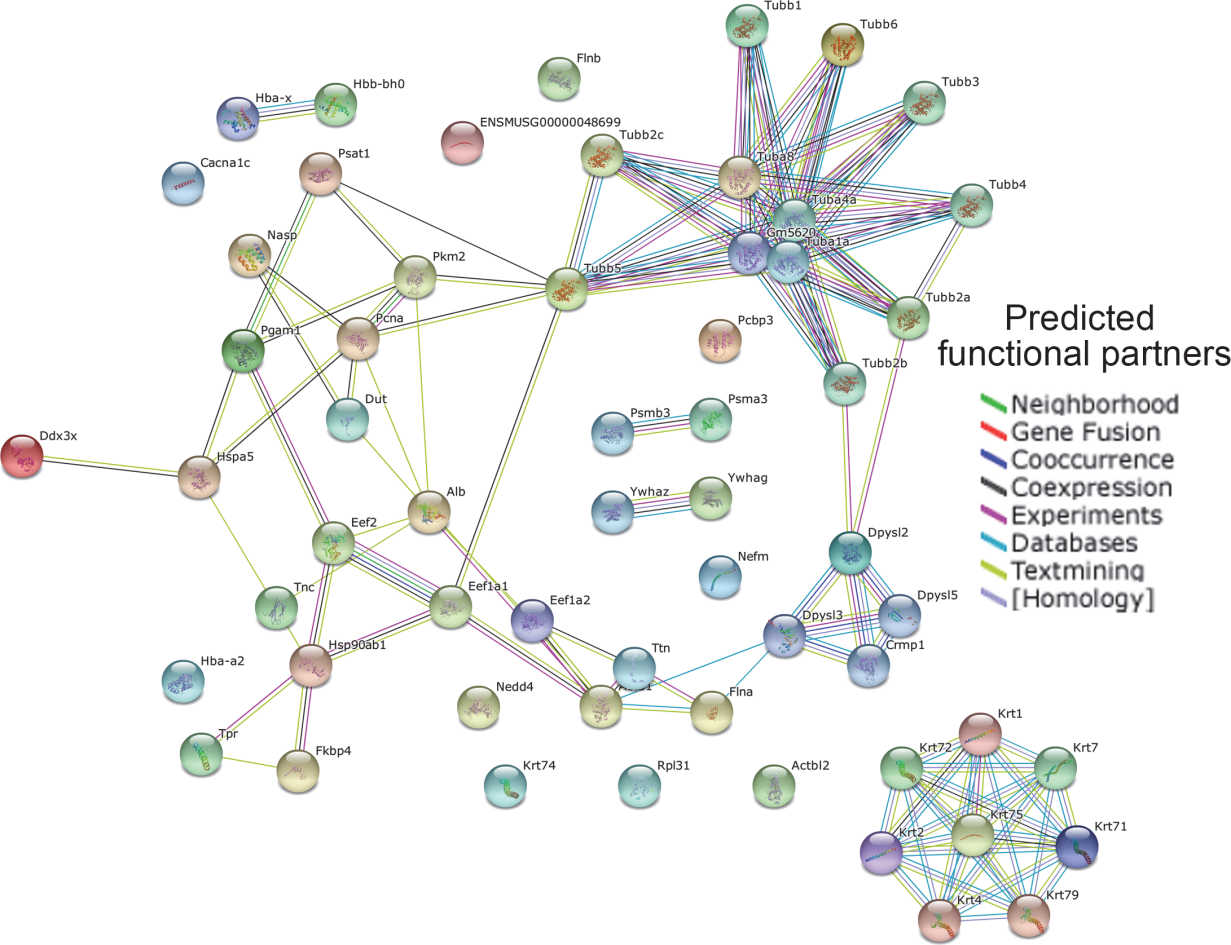
Functional protein association network of differentially expressed proteins in cortices at E13.5 and P1. Different line colors represent the types of evidence for the association: green, neighborhood; red, gene fusion; blue, co-occurrence; black, co-expression; purple, experiments; turquoise, database; yellow, text mining; and aqua, homology. The default setting for SPRING 9.1 was used to perform the analysis.

The differentially expressed proteins in cortices between E13.5 and P1 were observed to form two major clusters, which include 1) microtubule (Tubas and Tubbs), metabolism (Dpysl2, Dpysl3, Dpysl5, Crmp1 and others), and axonal development (Crmp1, Dpysl5, and Tnc), and 2) keratins (Krts) (Fig 6). Another small group of metabolism proteins (Psma3 and Psmb3) was coupled but some other proteins such as Pcbp3 and Cacna1c were not connected to any sub-network (Fig 6). These protein networks suggest that multiple proteins function cohesively in order to control a biological event such as metabolism in the developing cortex.

## Discussion

Proper development of the cerebral cortex requires precise protein expression regulation. To explore the protein expression signature in developing mouse cortices, we performed proteomic analysis on cortices of E13.5, E15.5 and P1 mice. Our results have shown protein expression profiles that mostly reflect important biological processes during cortical development. Differentially expressed proteins identified using proteomics can be used for further functional analyses.

Several studies have investigated protein expression in the brain. An early report resolved brain cytosol polymorphic proteins using the two-dimensional electrophoresis (2-DE) technology [11]. To increase the proteome coverage, a study examined proteomes of the adult mouse whole brain using combined global proteomic analysis and cysteinyl-peptide enrichment [12]. The proteome between neonatal and adult brains has been compared by using the 2-DE matrix-assisted laser desorption-mass spectrometry (MALDI)-time-of-flight (TOF)/TOF [13]. However, protein expression profiles of embryonic brains are not well understood. Furthermore, proteomes of several brain regions, including hippocampus, parietal cortex and cerebellum, from human adult brain samples have been analyzed by the isobaric tag for relative and absolute quantitation (iTRAQ)-based two-dimensional liquid chromatography coupled with tandem mass spectrometry (2D-LC-MS/MS). Using this strategy, reduced ubiquitin proteosome degradation system was identified as one of the causative factors for Alzheimer’s disease [14]. Region specific proteome from the adult mouse brain also was studied using the 2-DE-MALDI-TOF-MS technology [15]. Proteomes of whole brains from the embryonic stage and postnatal stage of Down syndrome model mice and wild type mice were compared using a 2-DE approach [16]. Until now, proteomes of the developing mouse brains, particularly the cerebral cortex, have not been thoroughly examined. Our study here has generated and compared protein expression profiles in mouse cortices at E13.5, E15.5 and P1.

In this study, we normalized and log2 transformed the proteomic data to make results from each sample comparable and the comparisons reliable. We detected a high number of proteins that are found in cortices of all three stages. A profile of such proteins suggests conserved functions that may maintain proper development in the cortex. For example, proteins regulating translation (Eif4a2, Rps5, and Rpl8) and metabolic process (Aldoc, Gapdhs, and Pgk2) have been identified with enriched expression in cortices at three stages. These results suggest that proper formation of the developing cortex requires active protein synthesis and metabolism as basic biology features. The higher ratio of differentially expressed proteins in cortices of E13.5 and P1 (3.5%) than that in cortices of E13.5 and E15.5 (2%) indicate that protein expression signatures in embryonic cortices are similar. These signatures reflect distinct developmental processes in cortices at embryonic and postnatal stages.

Functional pathway analyses have identified differential expression of proteins that regulate microtubule organization, cell communication (gap junction) and cell cycle in cortices at E13.5 and E15.5. For example, Cdk2 is an important cell cycle regulator that is essential for G1/S phase transition [26,27]. 14-4-3 isoforms bind to a wide spectrum of signal proteins, such as kinases, phosphatases and transmembrane receptors, and have been shown to regulate cell proliferation by interacting with the PI3 kinase and the FGF signaling [28–30]. Enriched expression of these proteins in the cortex indicates important embryonic stages of expansion of the progenitor pool through regulating the cell cycle. Furthermore, the STRING network analysis has identified interaction networks among differentially expressed proteins. For example, we have found that tubulin (Tubas and Tubbs), and keratin (Krts) isoforms form interactions, and 14-3-3 (Ywhae, Ywhag, and Ywhaz) are also connected to each other in the network. These network interactions will help further understand how various proteins function cohesively in controlling cortical development.

Our study has identified a group of proteins that are highly expressed in the mouse cortex at different developmental stages. We have revealed potential functions and crucial pathways of differentially expressed proteins in the cortex. Our study has provided evidences from proteome level to understand molecular mechanisms underlying cortical development. Further experimental studies should demonstrate protein interaction network in controlling proper formation of the cortex.

## Supporting Information

S1 Fig Hierarchical clustering for the developing cerebral cortexes. The mean of each protein expression value was calculated. Each protein expressed in the cortex at each stage was calculated and used to perform hierarchical clustering analysis.
(TIF)

S2 Fig Analyses of biological process (A), molecular function (B), and cellular component (C) for proteins differentially expressed in cortices between E13.5 and P1. The default setting for Database for Annotation, Visualization and Integrated Discovery (DAVID) was used to perform the analysis. Only top 10 enriched terms were shown in the pie chart.
(TIF)

S1 Table Proteins expressed in mouse cerebral cortices at E13.5, E15.5 and P1 identified using tandem mass spectrometry (MS/MS). (XLSX)

S2 Table Differentially expressed proteins identified in cortices between E13.5 and E15.5, and E13.5 and P1. (XLSX)

S3 Table Gene Ontology (GO) biological process analyses of proteins expressed in cortices of E13.5, E15.5 and P1 mice. (XLSX)

S4 Table Gene Ontology (GO) analyses of proteins differentially expressed in cortices between E13.5 and E15.5 mice. (XLSX)

S5 Table Gene Ontology (GO) analyses of proteins differentially expressed in cortices between E13.5 and P1 mice. (XLSX)

S6 Table KEGG pathway analyses of proteins differentially expressed between E13.5 and E15.5, and E13.5 and P1 mice. (XLSX)

## References

[1] Rakic P (2003) Developmental and evolutionary adaptations of cortical radial glia. Cereb Cortex 13: 541–549. 12764027 10.1093/cercor/13.6.541

[2] Götz M, Huttner WB (2005) The cell biology of neurogenesis. Nat Rev Mol Cell Biol 6: 777–788. 16314867 10.1038/nrm1739

[3] Noctor SC, Flint AC, Weissman TA, Dammerman RS, Kriegstein AR (2001) Neurons derived from radial glial cells establish radial units in neocortex. Nature 409: 714–720. 11217860 10.1038/35055553

[4] Haubensak W, Attardo A, Denk W, Huttner WB (2004) Neurons arise in the basal neuroepithelium of the early mammalian telencephalon: a major site of neurogenesis. Proc Natl Acad Sci U S A 101: 3196–3201. 14963232 10.1073/pnas.0308600100 PMC365766

[5] Chenn A, McConnell SK (1995) Cleavage orientation and the asymmetric inheritance of Notch1 immunoreactivity in mammalian neurogenesis. Cell 82: 631–641. 7664342 10.1016/0092-8674(95)90035-7

[6] Yang YT, Wang CL, Van Aelst L (2012) DOCK7 interacts with TACC3 to regulate interkinetic nuclear migration and cortical neurogenesis. Nat Neurosci. 10.1038/nn.3171 PMC3431462 22842144

[7] Englund C, Fink A, Lau C, Pham D, Daza RA, Bulfone A, et al (2005) Pax6, Tbr2, and Tbr1 are expressed sequentially by radial glia, intermediate progenitor cells, and postmitotic neurons in developing neocortex. J Neurosci 25: 247–251. 15634788 10.1523/JNEUROSCI.2899-04.2005 PMC6725189

[8] Guillemot F (2005) Cellular and molecular control of neurogenesis in the mammalian telencephalon. Curr Opin Cell Biol 17: 639–647. 16226447 10.1016/j.ceb.2005.09.006

[9] Molyneaux BJ, Arlotta P, Menezes JR, Macklis JD (2007) Neuronal subtype specification in the cerebral cortex. Nat Rev Neurosci 8: 427–437. 17514196 10.1038/nrn2151

[10] Wilhelm M, Schlegl J, Hahne H, Moghaddas Gholami A, Lieberenz M, Savitski MM, et al (2014) Mass-spectrometry-based draft of the human proteome. Nature 509: 582–587. 10.1038/nature13319 24870543

[11] Klose J, Nock C, Herrmann M, Stuhler K, Marcus K, Bluggel M, et al (2002) Genetic analysis of the mouse brain proteome. Nat Genet 30: 385–393. 11912495 10.1038/ng861

[12] Wang H, Qian WJ, Chin MH, Petyuk VA, Barry RC, Liu T, et al (2006) Characterization of the mouse brain proteome using global proteomic analysis complemented with cysteinyl-peptide enrichment. J Proteome Res 5: 361–369. 16457602 10.1021/pr0503681 PMC1850945

[13] Carrette O, Burkhard PR, Hochstrasser DF, Sanchez JC (2006) Age-related proteome analysis of the mouse brain: a 2-DE study. Proteomics 6: 4940–4949. 16912971 10.1002/pmic.200600084

[14] Manavalan A, Mishra M, Feng L, Sze SK, Akatsu H, Heese K (2013) Brain site-specific proteome changes in aging-related dementia. Exp Mol Med 45: e39 10.1038/emm.2013.76 24008896 PMC3789264

[15] Taraslia VK, Kouskoukis A, Anagnostopoulos AK, Stravopodis DJ, Margaritis LH, Tsangaris GT (2013) Proteomic analysis of normal murine brain parts. Cancer Genomics Proteomics 10: 125–154. 23741028

[16] Ishihara K, Kanai S, Sago H, Yamakawa K, Akiba S (2014) Comparative proteomic profiling reveals aberrant cell proliferation in the brain of embryonic Ts1Cje, a mouse model of Down syndrome. Neuroscience 281C: 1–15. 10.1016/j.neuroscience.2014.09.039 25261685

[17] Liu C, Zhang N, Yu H, Chen Y, Liang Y, Deng H, et al (2011) Proteomic analysis of human serum for finding pathogenic factors and potential biomarkers in preeclampsia. Placenta 32: 168–174. 10.1016/j.placenta.2010.11.007 21145106 PMC3039093

[18] Huang da W, Sherman BT, Lempicki RA (2009) Systematic and integrative analysis of large gene lists using DAVID bioinformatics resources. Nat Protoc 4: 44–57. 10.1038/nprot.2008.211 19131956

[19] Huang da W, Sherman BT, Lempicki RA (2009) Bioinformatics enrichment tools: paths toward the comprehensive functional analysis of large gene lists. Nucleic Acids Res 37: 1–13. 10.1093/nar/gkn923 19033363 PMC2615629

[20] Kanehisa M, Goto S, Sato Y, Kawashima M, Furumichi M, Tanabe M (2014) Data, information, knowledge and principle: back to metabolism in KEGG. Nucleic Acids Res 42: D199–205. 10.1093/nar/gkt1076 24214961 PMC3965122

[21] Kanehisa M, Goto S (2000) KEGG: kyoto encyclopedia of genes and genomes. Nucleic Acids Res 28: 27–30. 10592173 10.1093/nar/28.1.27 PMC102409

[22] Lian G, Lu J, Hu J, Zhang J, Cross SH, Ferland RJ, et al (2012) Filamin a regulates neural progenitor proliferation and cortical size through Wee1-dependent Cdk1 phosphorylation. J Neurosci 32: 7672–7684. 10.1523/JNEUROSCI.0894-12.2012 22649246 PMC3368379

[23] Tischfield MA, Baris HN, Wu C, Rudolph G, Van Maldergem L, He W, et al (2010) Human TUBB3 mutations perturb microtubule dynamics, kinesin interactions, and axon guidance. Cell 140: 74–87. 10.1016/j.cell.2009.12.011 20074521 PMC3164117

[24] Wu X, Rauch TA, Zhong X, Bennett WP, Latif F, Krex D, et al (2010) CpG island hypermethylation in human astrocytomas. Cancer Res 70: 2718–2727. 10.1158/0008-5472.CAN-09-3631 20233874 PMC2848870

[25] Franceschini A, Szklarczyk D, Frankild S, Kuhn M, Simonovic M, Roth A, et al (2013) STRING v9.1: protein-protein interaction networks, with increased coverage and integration. Nucleic Acids Res 41: D808–815. 10.1093/nar/gks1094 23203871 PMC3531103

[26] Pagano M, Pepperkok R, Lukas J, Baldin V, Ansorge W, Bartek J, et al (1993) Regulation of the cell cycle by the cdk2 protein kinase in cultured human fibroblasts. J Cell Biol 121: 101–111. 8458862 10.1083/jcb.121.1.101 PMC2119764

[27] Donjerkovic D, Scott DW (2000) Regulation of the G1 phase of the mammalian cell cycle. Cell Res 10: 1–16. 10765979 10.1038/sj.cr.7290031

[28] Radhakrishnan VM, Putnam CW, Martinez JD (2012) Activation of phosphatidylinositol 3-kinase (PI3K) and mitogen-activated protein kinase (MAPK) signaling and the consequent induction of transformation by overexpressed 14-3-3gamma protein require specific amino acids within 14-3-3gamma N-terminal variable region II. J Biol Chem 287: 43300–43311. 10.1074/jbc.M112.397877 23115241 PMC3527917

[29] Lonic A, Barry EF, Quach C, Kobe B, Saunders N, Guthridge MA (2008) Fibroblast growth factor receptor 2 phosphorylation on serine 779 couples to 14-3-3 and regulates cell survival and proliferation. Mol Cell Biol 28: 3372–3385. 10.1128/MCB.01837-07 18332103 PMC2423162

[30] Hermeking H, Benzinger A (2006) 14-3-3 proteins in cell cycle regulation. Semin Cancer Biol 16: 183–192. 16697662 10.1016/j.semcancer.2006.03.002

[31] Jablonska B, Aguirre A, Vandenbosch R, Belachew S, Berthet C, Kaldis P, et al (2007) Cdk2 is critical for proliferation and self-renewal of neural progenitor cells in the adult subventricular zone. J Cell Biol 179: 1231–1245. 18086919 10.1083/jcb.200702031 PMC2140044

[32] Kubben FJ, Peeters-Haesevoets A, Engels LG, Baeten CG, Schutte B, Arends JW, et al (1994) Proliferating cell nuclear antigen (PCNA): a new marker to study human colonic cell proliferation. Gut 35: 530–535. 7909785 10.1136/gut.35.4.530 PMC1374804

[33] Romaniello R, Tonelli A, Arrigoni F, Baschirotto C, Triulzi F, Bresolin N, et al (2012) A novel mutation in the β-tubulin gene TUBB2B associated with complex malformation of cortical development and deficits in axonal guidance. Dev Med Child Neurol 54: 765–769. 10.1111/j.1469-8749.2012.04316.x 22591407

[34] Mignon-Ravix C, Cacciagli P, El-Waly B, Moncla A, Milh M, Girard N, et al (2010) Deletion of YWHAE in a patient with periventricular heterotopias and pronounced corpus callosum hypoplasia. J Med Genet 47: 132–136. 10.1136/jmg.2009.069112 19635726

[35] Dai X, Jiang W, Zhang Q, Xu L, Geng P, Zhuang S, et al (2013) Requirement for integrin-linked kinase in neural crest migration and differentiation and outflow tract morphogenesis. BMC Biol 11: 107 10.1186/1741-7007-11-107 24131868 PMC3906977

[36] Ramachandran R, Reifler A, Parent JM, Goldman D (2010) Conditional gene expression and lineage tracing of tuba1a expressing cells during zebrafish development and retina regeneration. J Comp Neurol 518: 4196–4212. 10.1002/cne.22448 20878783 PMC2948409

[37] Breuss M, Heng JI, Poirier K, Tian G, Jaglin XH, Qu Z, et al (2012) Mutations in the β-tubulin gene TUBB5 cause microcephaly with structural brain abnormalities. Cell Rep 2: 1554–1562. 10.1016/j.celrep.2012.11.017 23246003 PMC3595605

[38] Ngo L, Haas M, Qu Z, Li SS, Zenker J, Teng KS, et al (2014) TUBB5 and its disease-associated mutations influence the terminal differentiation and dendritic spine densities of cerebral cortical neurons. Hum Mol Genet 23: 5147–5158. 10.1093/hmg/ddu238 24833723

[39] Abdollahi MR, Morrison E, Sirey T, Molnar Z, Hayward BE, Carr IM, et al (2009) Mutation of the variant alpha-tubulin TUBA8 results in polymicrogyria with optic nerve hypoplasia. Am J Hum Genet 85: 737–744. 10.1016/j.ajhg.2009.10.007 19896110 PMC2775839

[40] Middleton FA, Peng L, Lewis DA, Levitt P, Mirnics K (2005) Altered expression of 14-3-3 genes in the prefrontal cortex of subjects with schizophrenia. Neuropsychopharmacology 30: 974–983. 15726117 10.1038/sj.npp.1300674

[41] Wong AH, Macciardi F, Klempan T, Kawczynski W, Barr CL, Lakatoo S, et al (2003) Identification of candidate genes for psychosis in rat models, and possible association between schizophrenia and the 14-3-3eta gene. Mol Psychiatry 8: 156–166. 12610648 10.1038/sj.mp.4001237

[42] Cheah PS, Ramshaw HS, Thomas PQ, Toyo-Oka K, Xu X, Martin S, et al (2012) Neurodevelopmental and neuropsychiatric behaviour defects arise from 14-3-3ζ deficiency. Mol Psychiatry 17: 451–466. 10.1038/mp.2011.158 22124272

[43] von Holst A, Egbers U, Prochiantz A, Faissner A (2007) Neural stem/progenitor cells express 20 tenascin C isoforms that are differentially regulated by Pax6. J Biol Chem 282: 9172–9181. 17264084 10.1074/jbc.M608067200

[44] Cushion TD, Paciorkowski AR, Pilz DT, Mullins JG, Seltzer LE, Marion RW, et al (2014) De novo mutations in the beta-tubulin gene TUBB2A cause simplified gyral patterning and infantile-onset epilepsy. Am J Hum Genet 94: 634–641. 10.1016/j.ajhg.2014.03.009 24702957 PMC3980418

[45] Ozeki Y, Pickard BS, Kano S, Malloy MP, Zeledon M, Sun DQ, et al (2011) A novel balanced chromosomal translocation found in subjects with schizophrenia and schizotypal personality disorder: altered l-serine level associated with disruption of PSAT1 gene expression. Neurosci Res 69: 154–160. 10.1016/j.neures.2010.10.003 20955740 PMC3049551

[46] Yamashita N, Uchida Y, Ohshima T, Hirai S, Nakamura F, Taniguchi M, et al (2006) Collapsin response mediator protein 1 mediates reelin signaling in cortical neuronal migration. J Neurosci 26: 13357–13362. 17182786 10.1523/JNEUROSCI.4276-06.2006 PMC6674993

[47] Drinjakovic J, Jung H, Campbell DS, Strochlic L, Dwivedy A, Holt CE (2010) E3 ligase Nedd4 promotes axon branching by downregulating PTEN. Neuron 65: 341–357. 10.1016/j.neuron.2010.01.017 20159448 PMC2862300

[48] Burger C, Lopez MC, Baker HV, Mandel RJ, Muzyczka N (2008) Genome-wide analysis of aging and learning-related genes in the hippocampal dentate gyrus. Neurobiol Learn Mem 89: 379–396. 10.1016/j.nlm.2007.11.006 18234529 PMC2530823

[49] Tanaka H, Morimura R, Ohshima T (2012) Dpysl2 (CRMP2) and Dpysl3 (CRMP4) phosphorylation by Cdk5 and DYRK2 is required for proper positioning of Rohon-Beard neurons and neural crest cells during neurulation in zebrafish. Dev Biol 370: 223–236. 10.1016/j.ydbio.2012.07.032 22898304

[50] Morimura R, Nozawa K, Tanaka H, Ohshima T (2013) Phosphorylation of Dpsyl2 (CRMP2) and Dpsyl3 (CRMP4) is required for positioning of caudal primary motor neurons in the zebrafish spinal cord. Dev Neurobiol 73: 911–920. 10.1002/dneu.22117 23929741

[51] Richter F, Meurers BH, Zhu C, Medvedeva VP, Chesselet MF (2009) Neurons express hemoglobin α- and β-chains in rat and human brains. J Comp Neurol 515: 538–547. 10.1002/cne.22062 19479992 PMC3123135

[52] Sutton MA, Taylor AM, Ito HT, Pham A, Schuman EM (2007) Postsynaptic decoding of neural activity: eEF2 as a biochemical sensor coupling miniature synaptic transmission to local protein synthesis. Neuron 55: 648–661. 17698016 10.1016/j.neuron.2007.07.030

[53] Lee AS, Ra S, Rajadhyaksha AM, Britt JK, De Jesus-Cortes H, Gonzales KL, et al (2012) Forebrain elimination of cacna1c mediates anxiety-like behavior in mice. Mol Psychiatry 17: 1054–1055. 10.1038/mp.2012.71 22665262 PMC3481072

[54] Nakajima J, Okamoto N, Tohyama J, Kato M, Arai H, Funahashi O, et al. (2014) De novo EEF1A2 mutations in patients with characteristic facial features, intellectual disability, autistic behaviors and epilepsy. Clin Genet. 10.1111/cge.12394 24697219

