# Supplementary figures and images for: Protein Expression Profiles Characterize Distinct Features of Mouse Cerebral Cortices at Different Developmental Stages
Source: PLoS One. 2015 Apr 27;10(4):e0125608. doi: 10.1371/journal.pone.0125608 (PMC4411115; doi:10.1371/journal.pone.0125608)

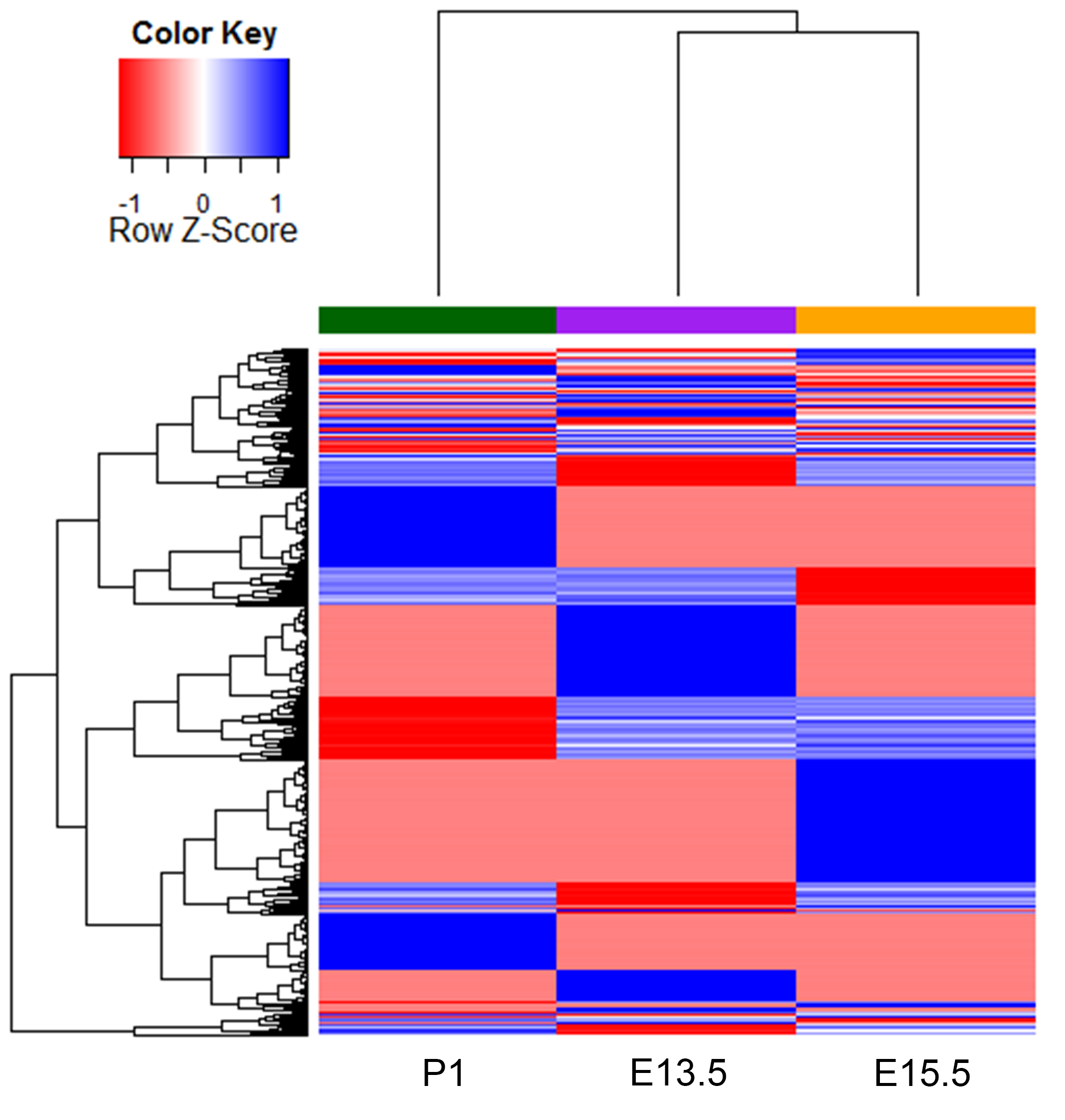

Supplement: S1 Fig — The mean of each protein expression value was calculated. Each protein expressed in the cortex at each stage was calculated and used to perform hierarchical clustering analysis. (TIF) [file pone.0125608.s001.tif]

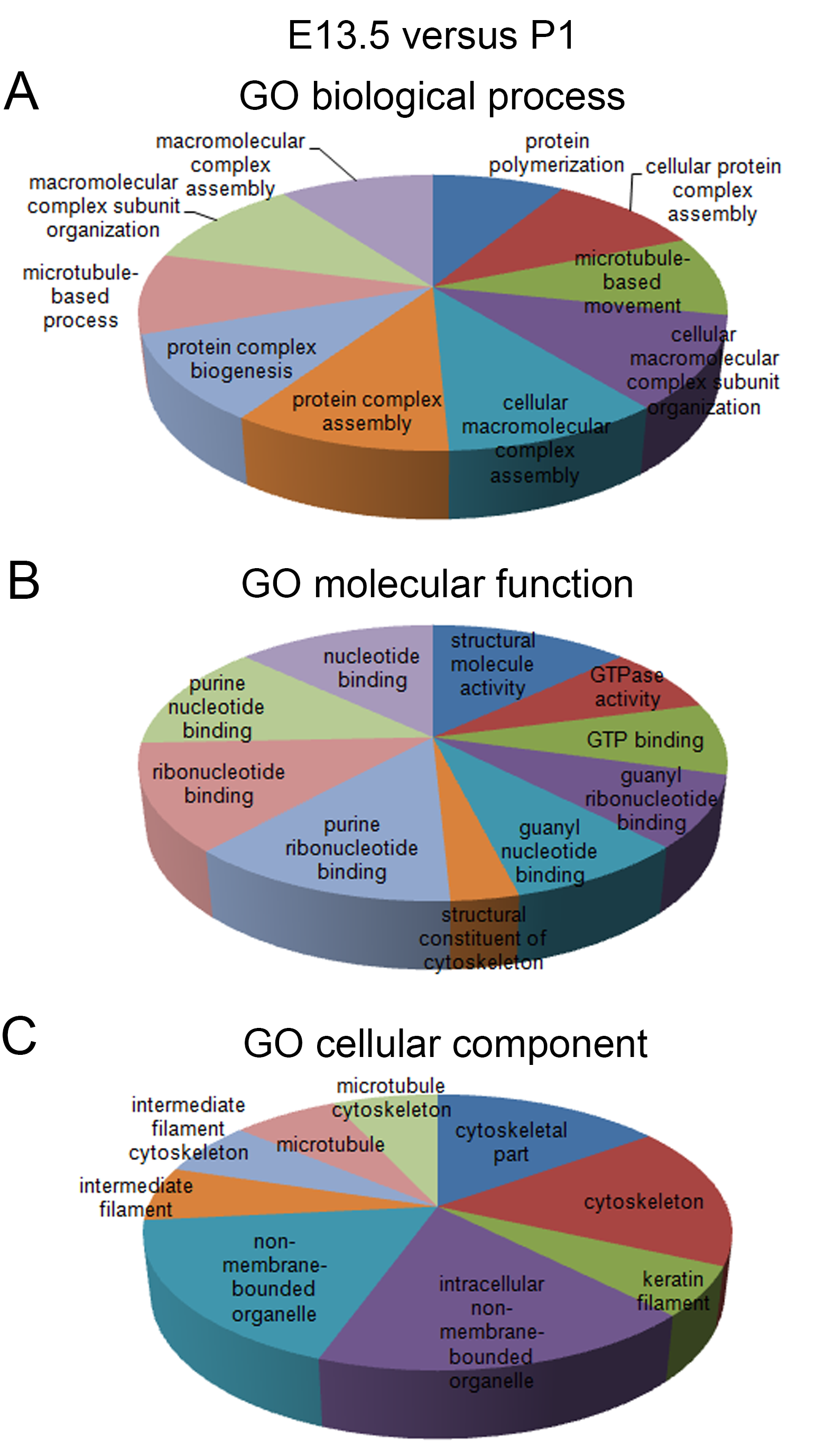

Supplement: S2 Fig — The default setting for Database for Annotation, Visualization and Integrated Discovery (DAVID) was used to perform the analysis. Only top 10 enriched terms were shown in the pie chart. (TIF) [file pone.0125608.s002.tif]
